# ECDD-S16, a synthetic derivative of cleistanthin A, suppresses pyroptosis in *Burkholderia pseudomallei-*infected U937 macrophages

**DOI:** 10.1371/journal.pone.0327457

**Published:** 2025-07-08

**Authors:** Suphasuta Khongpraphan, Sucharat Sanongkiet, Chularat Luangjindarat, Sarut Thairat, Bumrung Munyoo, Napason Chabang, Sitthivut Charoensutthivarakul, Suparerk Borwornpinyo, Patoomratana Tuchinda, Marisa Ponpuak, Pongsak Utaisincharoen, Matsayapan Pudla

**Affiliations:** 1 Department of Microbiology, Faculty of Science, Mahidol University, Bangkok, Thailand; 2 Mahidol Oxford Tropical Medicine Research Unit, Faculty of Tropical Medicine, Mahidol University, Bangkok, Thailand; 3 Department of Chemistry, Faculty of Science, Silpakorn University, Nakhon Pathom, Thailand; 4 Division of Medical Bioinformatics, Research Department, Faculty of Medicine Siriraj Hospital, Mahidol University, Bangkok, Thailand; 5 Oral Tissues, Cells and Molecular Biology Analysis and Research Center, Faculty of Dentistry, Mahidol University, Bangkok, Thailand; 6 Excellence Center for Drug Discovery (ECDD), Faculty of Science, Mahidol University, Bangkok, Thailand; 7 School of Bioinnovation and Bio-Based Product Intelligence, Faculty of Science, Mahidol University, Bangkok, Thailand; 8 Center for Neuroscience, Faculty of Science, Mahidol University, Bangkok, Thailand; 9 Department of Biotechnology, Faculty of Science, Mahidol University, Bangkok, Thailand; 10 Department of Oral Microbiology, Faculty of Dentistry, Mahidol University, Bangkok, Thailand; University of Oxford, UNITED KINGDOM OF GREAT BRITAIN AND NORTHERN IRELAND

## Abstract

**Background:**

Melioidosis is an infectious disease caused by an intracellular Gram-negative bacterium, *Burkholderia pseudomallei,* which is a common cause of community-acquired sepsis in Southeast Asia and Northern Australia. The mortality rate in acute melioidosis patients, which is caused by sepsis, is very high (47.1%). Therefore, reducing inflammation may lead to the treatment of patients with acute melioidosis. Previously, ECDD-S16 was reported to be a potential compound for inhibiting inflammatory cell death (pyroptosis).

**Objective:**

In this study, we further investigated the involvement of ECDD-S16 in pyroptosis induced by *B. pseudomallei* in the U937 human macrophage cell line.

**Methods:**

To investigate the biological activity of ECDD-S16, U937 macrophages were infected with *B. pseudomallei* before treatment with the compound. The expression of pyroptosis marker was determined by lactate dehydrogenase (LDH) assay, western blotting and ELISA assay. Additionally, the intracellular growth of *B. pseudomallei* was examined by CFU determination. Furthermore, colocalization of the bacteria with phagosome acidification was observed by immunofluorescent staining.

**Results:**

The results showed that ECDD-S16 decreased LDH release and levels of pyroptosis-related proteins in *B. pseudomallei*-infected cells by inhibiting phagolysosome acidification. Moreover, the attenuation of pyroptosis did not interfere with the intracellular survival of *B. pseudomallei* in U937 macrophages.

**Conclusion:**

Our findings indicated that ECDD-S16, a novel compound, interferes with caspase-1/4/5 activation, which may lead to the prevention of sepsis in acute melioidosis patients.

## Introduction

Melioidosis is a multifaceted infectious disease caused by the Gram-negative intracellular bacterium *Burkholderia pseudomallei* [1]. One of the pathogenesis hallmarks of this bacterium is the ability to adopt an intracellular lifestyle within phagocytes [2]. Following the host cell invasion, *B. pseudomallei* residing within the phagosome resists host cell killing mechanisms using a type 3 secretion system (T3SS), leading to cell-to-cell protrusion and induction of multinucleated giant cells (MNGCs) [3,4]. However, the expression of multiple virulence factors at various stages of intracellular infection allows rapid disease progression [1]. There are around 165,000 cases of human melioidosis per year globally and mortality rate due to melioidosis was estimated to be up to 89,000 deaths annually [5]. In Thailand, melioidosis cases are prevalent mostly in the Northeastern provinces [6]. The disease manifestation appears as abscess formation in several organs, such as the lung, liver, spleen and soft tissues [7]. Moreover, this manifestation is associated with acute or chronic diseases and can lead to fatal sepsis. Infection leading to sepsis is considered one of the biggest health problems with this disease worldwide, leading to the dysregulation of inflammation and immune responses [8].

Pyroptosis, an inflammatory cell death pathway, plays an essential role in fighting against infection. Excessive pyroptosis can lead to immune desensitization and sepsis [9,10]. This type of cell death is defined as gasdermin-mediated programmed cell death and is a part of innate immune response that can be triggered by canonical and non-canonical inflammasome activation [11]. Upon activation, it can induce maturation of caspase-1 (canonical inflammasome activation) or caspase-4/5 (non-canonical inflammasome activation), both of which can cleave the pore-forming protein gasdermin D (GSDMD) into GSDMD-NT leading to cell membrane rupture and massive release of inflammatory mediators, including IL-1β, IL-18, ATP and LDH, causing severe inflammatory responses [12]. There is growing evidence that, during the infection, macrophages are one of the main cells that produce proinflammatory cytokines, including IL-1β, IL-18, IL-6, and TNF-α. Since proinflammatory cytokines play a pivotal part in eradicating the bacteria, suppressing proinflammatory cytokines might lead to increased bacterial replication. Therefore, finding a compound that could attenuate proinflammatory cytokines during the infection without interfering with bacterial survival may be an alternative for treating acute melioidosis. Previously, our group demonstrated that ECDD-S16, a 4-fluorobenzoyl ester of cleistanthin A, exhibited a high potency in inhibiting pyroptosis induced by TLR ligands-activated RAW 264.7 macrophages [13]. Therefore, we further investigate the anti-inflammatory effect of ECDD-S16 on *B. pseudomallei*-infected U937 macrophages by focusing on pyroptosis activation and intracellular bacterial survival. This information may offer a potential candidate for drug development in searching for a novel compound to prevent overstimulation of pyroptosis induced by *B. pseudomallei* infection.

## Materials and methods

### Synthesis of ECDD-S16 and structure characterization

ECDD-S16 was acquired according to the procedure described previously [13]. To synthesize ECDD-S16, 4-fluorobenzoic acid (1.5 eq) was dissolved in dichloromethane under nitrogen gas. N,N′-Dicyclohexylcarbodiimide (DCC) (1.5 eq) was added, stirring the reaction for 5 minutes. CA (1.0 eq) and 4-dimethylaminopyridine (DMAP) (catalytic amount) was then added to the reaction and the mixture was left overnight at room temperature. When completed, the reaction was diluted with ethyl acetate and washed with saturated NH4Cl (aq.), water, brine, and dried over MgSO4. The organic portion was filtered and concentrated to give a crude product. Purification was performed using flash column chromatography over silica gel to obtain the desired product ECDD-S16 as off-white powder (36% yield). ECDD-S16 (1); 1H NMR (500 MHz, CDCl3): 8.11 (dd, J = 8.8, 5.4 Hz, 2H), 7.56 (d, J = 3.6 Hz, 1H), 7.14 (br t, J = 8.6 Hz, 2H), 7.01 (s, 1H), 6.94 (dd, J = 7.9, 1.0 Hz, 1H), 6.80 (dd, J = 5.0, 1.4 Hz, 1H), 6.78–6.75 (m, 1H), 6.01 (d, J = 0.9 Hz, 1H), 6.04 (d, J = 1.4 Hz, 1H), 5.58 (t, J = 6.8 Hz, 1H), 5.46 (dd, J = 14.5, 1.6 Hz, 1H), 5.38 (dd, J = 14.6, 4.3 Hz, 1H), 5.23 (dd, J = 6.1, 0.9 Hz, 1H), 4.23 (dd, J = 12.1, 3.4 Hz, 1H), 3.88 (s, 3H), 3.77 (s, 3H), 3.60 (s, 3H), 3.54 (s, 3H), 3.44 (dd, J = 11.8, 7.0 Hz, 1H). 13C NMR (500 MHz, CDCl3): 169.5, 166.1 (C-F, 1JC-F = 253.8 Hz), 164.5, 151.7, 150.2, 147.4, 143.9, 135.6, 132.4 (C-F, 3JC-F = 8.8 Hz), 130.5, 128.3, 126.4 (C-F, 4JC-F = 7.5 Hz), 125.6, 123.50, 119.2, 115.9 (C-F, 2JC-F = 21.3 Hz), 110.6, 108.2, 105.9, 101.2, 100.7, 81.2, 77.9, 72.0, 67.0, 62.7, 60.1, 58.5, 56.0, 55.8. 19F NMR (376 MHz, CDCl3): −104.0 (s). ESI-MS m/z: 663.1879 [M + H]+, (calcd. for C35H32FO12, 663.1878).

### Chemical reagents

A selective caspase-1 inhibitor (Ac-YVAD-cmk; SML0429) and caspase-4 inhibitor (Z-LEVD-FMK; ab120489) were purchased from Sigma-Aldrich and Abcam, respectively.

### Bacterial strains and culture conditions

*B. pseudomallei* strain 1026b was cultured in Luria-Bertani (LB) broth at 37°C with agitation at 150 rpm. Overnight cultures were washed twice in phosphate-buffered saline (PBS) and adjusted to a desired concentration by measuring the optical density at 650 nm. For colony forming units (CFU) determination, bacteria were plated on tryptic soy agar and incubated at 37 °C with aeration for 48 h.

### U937 cell culture and differentiation

U937 (ATCC CRL-1593.2), a human monocyte-like cell line was used in this study. The suspension cells were grown in RPMI (Hyclone, Utah, USA) supplemented with 10% FBS (Hyclone), 1% L-glutamine (Gibco Labs, Grand Island, NY, USA) at 37 °C, 5% CO_2_. For the assay, U937 cells were seeded at 1.5 × 10^6^ cells/well in a 6-well plate (Corning, New York, USA) and differentiated into macrophage-like cells by the addition of 10 ng/ml PMA (Phorbol 12-myristate 13-acetate, Sigma-Aldrich, Missouri, USA) for overnight at 37 °C, 5% CO_2_. PMA was removed after incubating overnight, followed by 2 days of rest before infection with *B. pseudomallei.*

### U937 macrophages infected with *B. pseudomallei*

U937 macrophages (1.5 x 10^6^ cells/well) were co-cultured with *B. pseudomallei* at multiplicity of infection (MOI) of 10 for 1 h. The cells were washed twice with 1 mL of PBS to remove extracellular bacteria and residual bacteria were killed by incubating the cells in RPMI containing 250 μg/ml kanamycin (Gibco) for 2 h and were allowed to continue until the experiment was terminated. The cell lysates were analyzed by Western blot and the supernatant of infected cells was analyzed by LDH and cytokine assay.

### LDH assay

The release of LDH in the culture supernatants was measured by cytotoxicity assay to determine the level of pyroptosis [14]. At the indicated time intervals, the supernatants of infected cells were collected and LDH activity was detected using the CytoTox 96^®^ non-radioactive cytotoxicity assay (Promega, Wisconsin, USA) according to the manufacturer’s instructions. The supernatants were applied to each well of the flat-bottom 96-well plate (NUNC^TM^ Brand Product). The substrate mix was then added and incubated at room temperature in the dark for 20 min followed by addition of the stop solution. The LDH measurements recorded absorbance at 490 nm (Ao Microplate reader, Azure biosystems Inc., Model AC3000). For maximum LDH release, 10X lysis solution was added to the uninfected cells and LDH detection was performed in the same manner. The percentage of LDH release was calculated using the following formula: % LDH release = (Experimental LDH release – Spontaneous LDH release/Maximum LDH release) x100 [14].

### Western blot analysis

The infected cells were lyzed in a lysis buffer containing 20 mM Tris, 100 mM NaCl and 1% NP-40. The lysates were separated on 15% SDS-PAGE gels. Proteins were transferred onto a nitrocellulose membrane (Amersham Biosciences, UK). First, the non-specific binding sites on the membrane were blocked with 3% BSA (Merck Millipore, Darmstadt, Germany) in TBST for 1 h before proteins were allowed to react with specific primary antibodies against caspase-1 (Cell Signaling; #2225), caspase-4 (Cell Signaling; #4450), cleaved-GSDMD (Cell Signaling; #36425), caspase-5 (Santa Cruz; sc-393346), cathepsin D (Abcam; ab75852) and actin (Merck Millipore; MAB1501) at 4°C overnight. Next, the membranes were washed three times with 0.1% PBST and incubated with horseradish peroxidase-conjugated goat anti-rabbit IgG or goat anti-mouse IgG (R&D Systems, Minnesota, USA) for 1 h at room temperature. Thereafter, the membranes were washed four times with 0.1% PBST before a chemiluminescence substrate (Roche Diagnostics, Basel, Switzerland) was added and protein bands were detected by enhanced chemiluminescence.

### ELISA for cytokine secretion

The supernatants from infected cells were collected. Cytokine production levels were measured by using Human IL-1β DuoSet ELISA, Human Total IL-18 DuoSet ELISA, Human IL-10 DuoSet ELISA (R&D Systems) and Human TNF ELISA Set (BD Biosciences) following the manufacturer’s instructions.

### MTT cell viability assay

Briefly, U937 monocyte-like cells (1.75 × 10^4^ cells/well) were seeded onto a flat-bottom 96-well plate (Corning) and differentiated into macrophage-like cells as described above in cell line and culture condition. The cells were treated with various concentrations of ECDD-S16 for 24 h. The cell viability was determined by MTT assay using the 3-(4,5-dimethylthiazol-2-yl)-2,5-diphenyltetrazolium bromide (MTT; Sigma-Aldrich). The absorbance of the solution was measured at 540 nm using a microplate reader (Ao Microplate reader, Azure biosystems Inc., Model AC3000). The percent cell viability was calculated by using % cell viability = [(Absorbance of treated cells – Absorbance of blank)/(Absorbance of DMSO control cells – Absorbance of blank)] × 100.

### Lysotracker and immunofluorescence staining of phagolysosomal compartments

For staining and co-localization analysis of acidic vacuoles with *B. pseudomallei*, U937 monocyte-like cells (4× 10^5^ cells/well) were seeded on a sterile glass coverslip in a 24-well plate (Corning) and differentiated into macrophage-like cells as described above. After incubation, the cells were infected with *B. pseudomallei* at an MOI of 10. Following this, the bacteria-containing medium was discarded, the RPMI containing kanamycin was added and incubated at 37 °C, 5% CO_2_ to remove extracellular bacteria. The acidic vacuoles of the cells were labeled with LysoTracker (Molecular probes, Life Technologies) for at least 30 min at 37°C before fixation with 4% paraformaldehyde for 5 min. Cells were then permeabilized and blocked with 0.1% saponin (Sigma-Aldrich) diluted in 3% bovine serum albumin (BSA) (Merck Millopore)/PBS for 1 h. For LAMP-1 staining, the infected cells were fixed with 4% paraformaldehyde for 5 min before permeabilized and blocked, as described above. Next, the permeabilized cells were processed for anti-LAMP-1 (1:200 dilution, Proteintech; PE-65051) for 2 h. Cells were then washed three times with PBS and subsequently incubated with anti-*B. pseudomallei* (4B11 monoclonal antibody) labeled with Alexa Fluor 488 fluorescent dye for 1 h. The stained cells were then washed three times with PBS. The nucleus was stained with Hoechst 33342 (Thermo Fisher Scientific) for 15 min. The cells were washed three times with PBS and mounted on the glass slides using ProLong Gold antifade reagent (Invitrogen, Massachusetts, USA). Stained cells and bacteria were imaged by confocal laser scanning microscopy (Leica Stellaris 5).

### Statistical analysis

All experiments were performed at least three independent times. The results were expressed as mean ± SEM. All data were analyzed by the Prism software (GraphPad) by using the Student’s t-test or one-way ANOVA test followed by a *post-hoc* multiple comparison test based on specific experiments. Asterisks indicate statistically significant differences based on p-values: * for *p* < 0.05, ** for *p* < 0.01, *** for *p* < 0.001 and **** for *p* < 0.0001.

## Results

### ECDD-S16 suppresses caspase activation in *B. pseudomallei*-infected U937 macrophages

**Fig 1:**
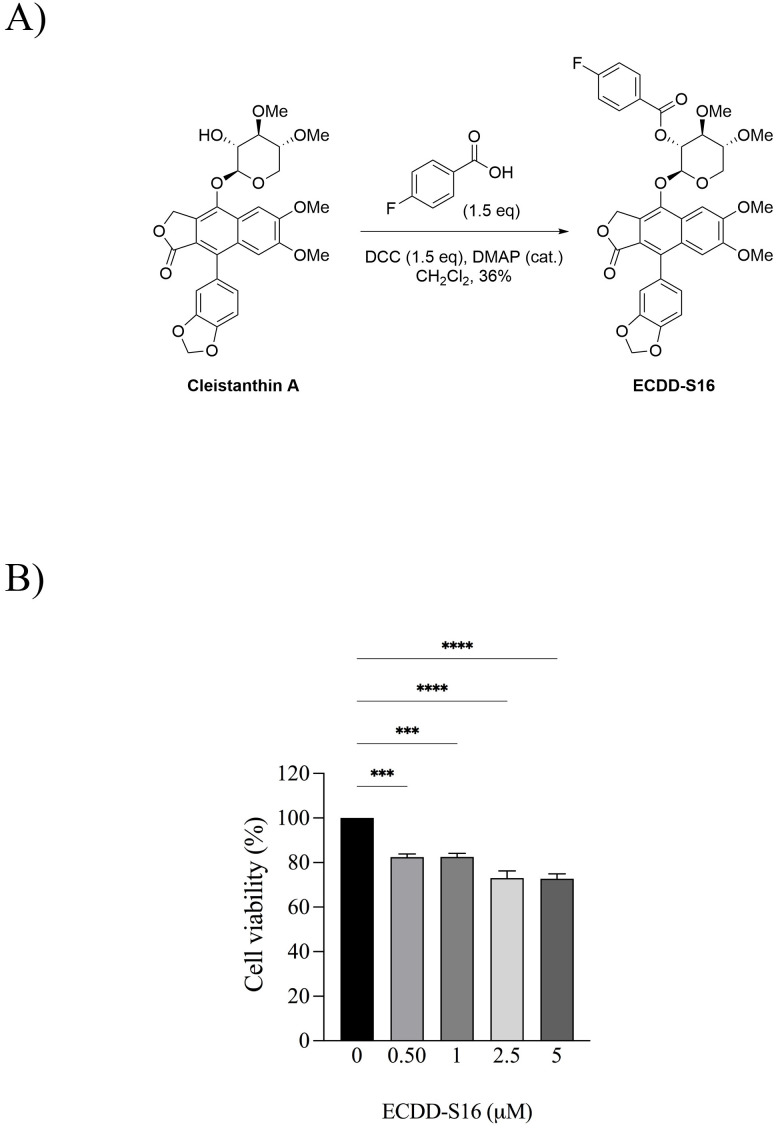
Chemical structure of ECDD-S16 and the cytotoxicity effect on U937 macrophages. (A) ECDD-S16 is modified from the esterification of cleistanthin A with 4-fluorobenzoic acid in the presence of N,N′-dicyclohexylcarbodiimide (DCC) and a catalytic amount of 4-dimethylaminopyridine (DMAP). (B) The cells were exposed to ECDD-S16 at concentrations ranging from 0 to 5 μM for 24 h. The viability of the treated cells was analyzed by an MTT assay. Data are expressed as mean ± SEM from three independent experiments. One-way ANOVA followed by Tukey’s multiple comparison tests was used to compare the cell viability. ***, *P* < 0.001, ****, *P* < 0.0001.

**Fig 2:**
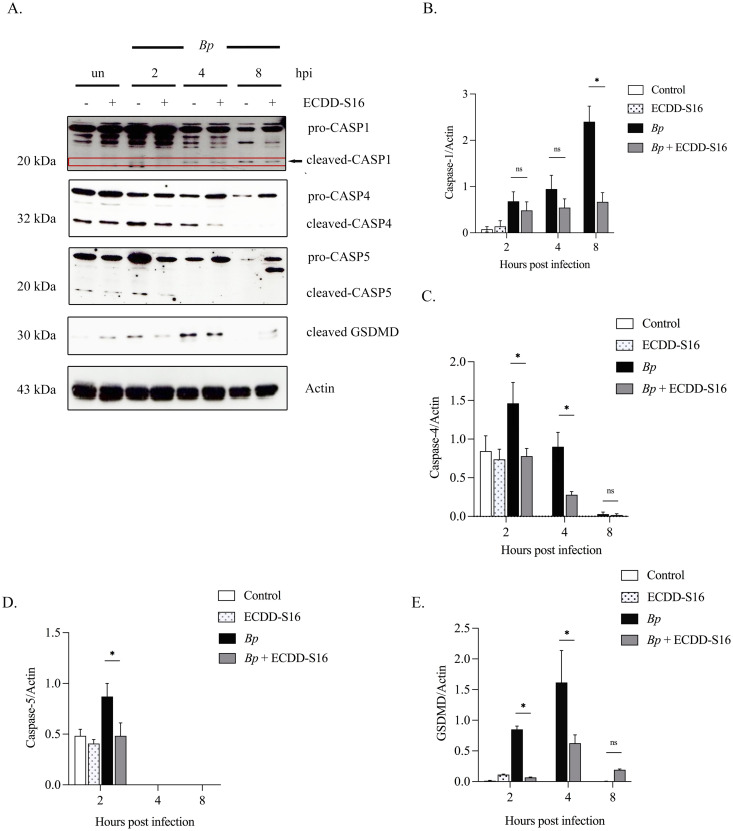
ECDD-S16 prevents activation of caspase-1, caspase-4 and caspase-5 cleavages in *B. pseudomallei*-infected U937 macrophages. The macrophages were infected with *B. pseudomallei* at an MOI of 10 for 1 h before ECDD-S16 (1 μM) treatment. Expression of caspases was determined by immunoblotting. (A) One representative image of at least three independent experiments is shown for Western blotting and **(B-E)** quantitative analysis of caspase-1/4/5 and gasdermin D by ImageJ. Data are expressed as mean ± SEM from three independent experiments. One-way ANOVA followed by Sidak’s multiple comparison test was used to compare the quantification of protein expression. *, *P* < 0.05, ns = not significant, un = uninfected cells, hpi = hours post-infection.

ECDD-S16 was synthesized by esterification of cleistanthin A (CA) with 4-fluorobenzoic acid (Fig 1A). To determine the cytotoxicity of this compound, U937 macrophages were treated with different concentrations of ECDD-S16, and the cytotoxicity was analyzed by MTT assay. It should be noted that the viability of the cells treated with ECDD-S16 at a concentration of 0.5 μM was greater than 80% and the IC_50_ is more than 5 μM (Fig 1B). However, in *B. pseudomallei-*infected U937 macrophages, the maximum time point was 8 h, and the cell viability of the infected cells at this timepoint was greater than 90% at the concentration of ECDD-S16 used (1 μM). Previously, our group demonstrated that ECDD-S16 exhibits the ability to inhibit pyroptosis induced by both surface and endosomal TLR ligands in RAW 264.7 cells [13]. The present study aimed to further evaluate whether ECDD-S16 could interfere with pyroptosis in *B. pseudomallei-*infected U937 macrophages. As shown in Fig 2A and 2B, the activation of caspase-1, the canonical inflammasome’s effector molecule, was decreased in ECDD-S16 treated cells at different time intervals. Additionally, caspase-4/5 (non-canonical inflammasome) attenuation was correlated with caspase-1 activation (Fig 2A, 2C and 2D). As expected, the decreased level of GSDMD cleavage (GSDMD-NT) was associated with suppressing caspase-1 and caspase-4/5-dependent pyroptosis (Fig 2A and 2E). These results implied that ECDD-S16 decreased the activation of caspase-1/4/5 in response to *B. pseudomallei* infection by preventing the cleavage of pro-caspase-1/4/5 in U937 macrophages. Therefore, the levels of the active form of these caspases were reduced.

### ECDD-S16 impairs the induction of pyroptosis in *B. pseudomallei*-infected U937 macrophages

**Fig 3:**
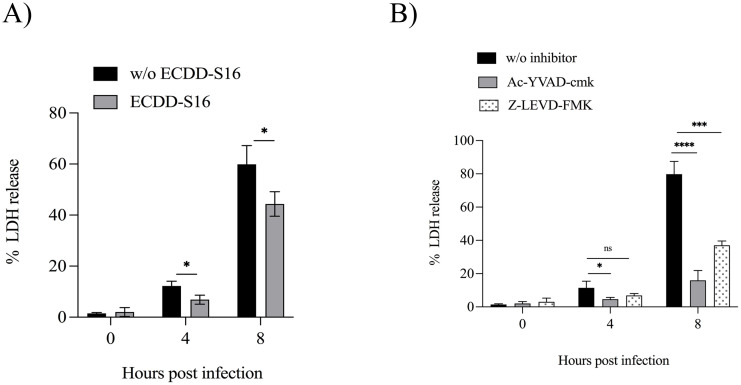
LDH release is influenced by caspase activation. (A) U937 macrophages were infected with *B. pseudomallei* at an MOI of 10 for 1 h followed by treatment with ECDD-S16 (1 μM). The percentage of lactate dehydrogenase (LDH) release in the infected cells was measured using LDH assay. (B) U937 macrophages were pretreated with 25 μM Ac-YVAD-cmk (caspase-1 inhibitor) for 30 min or 10 μM Z-LEVD-FMK (caspase-4 inhibitor) for 1 h before infection with *B. pseudomallei* at an MOI of 10. The percentage of lactate dehydrogenase (LDH) release in the infected cells was measured using LDH assay in the presence of these inhibitors. Data are expressed as mean ± SEM from three independent experiments. The Student’s t-test and One-way ANOVA followed by Tukey’s multiple comparison tests were used to compare LDH release. *, *P* < 0.05, ***, *P* < 0.001, ****, *P* < 0.0001, ns = not significant.

Since caspase-1/4/5 was associated with pyroptosis, next, we investigated whether the inhibition of these caspases by ECDD-S16 could affect the *B. pseudomallei-*induced pyroptosis in U937 macrophages by using LDH release as a marker. Fig 3A showed that the infected cells treated with ECDD-S16 were significantly impaired. To verify that the decrease in LDH release was directly related to the inhibition of these caspases, we pretreated the cells with 25 μM Ac-YVAC-cmk (caspase-1 inhibitor) for 30 min or 10 μM Z-LEVD-FMK (caspase-4 inhibitor) for 1 h before the infection. As shown in Fig 3B, the level of LDH release was significantly inhibited in the caspase-1 inhibitor-treated cells at 4 and 8 hpi. In contrast, in the caspase-4 inhibitor-treated cells, a significant reduction in this mediator at 8 hpi was observed (Fig 3B). These data suggested that ECDD-S16 inhibited *B. pseudomallei*-induced pyroptosis via caspase activation.

### ECDD-S16 only inhibits the production of IL-1βand IL-18 but not TNF-α and IL-10

**Fig 4:**
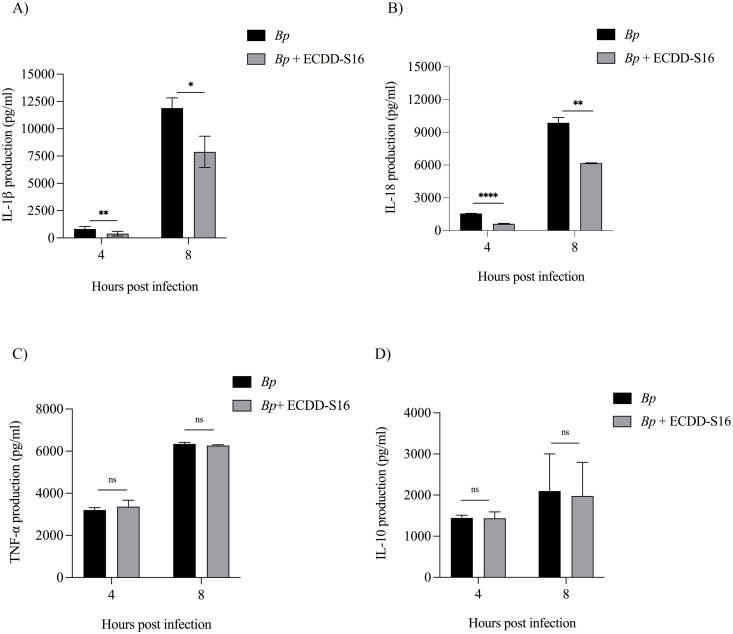
Interfering of cytokine production by ECDD-S16 in *B. pseudomallei-*infected U937 macrophages. (A-D) The cells were infected with *B. pseudomallei* at an MOI of 10 for 1 h before treatment with 1 μM ECDD-S16. At the indicated time points, the levels of cytokine production were determined by ELISA. Data are expressed as mean ± SEM from three independent experiments. The Student’s t-test was used to compare cytokine production. *, *P* < 0.05, **, *P* < 0.01, ****, *P* < 0.0001, ns = not significant.

One of the critical markers of pyroptosis is the release of IL-1β and IL-18. We therefore analyzed the effect of ECDD-S16 on the release of these inflammasome-dependent cytokines in *B. pseudomallei*-infected U937 macrophages. As expected, the production of IL-1β and IL-18 was significantly impaired in the ECDD-S16-treated cells (Fig 4A and 4B). Interestingly, the inflammatory response-related cytokines such as TNF-α or anti-inflammatory response-related cytokines such as IL-10, did not differ in the presence of this compound (Fig 4C and 4D). These results indicated an essential role of ECDD-S16 only in suppressing pyroptosis-related cytokines, particularly IL-1β and IL-18.

### Caspase inhibitors suppress IL-1β,IL-18 and IL-10 but not TNF-α in response to *B. pseudomallei* infection

**Fig 5:**
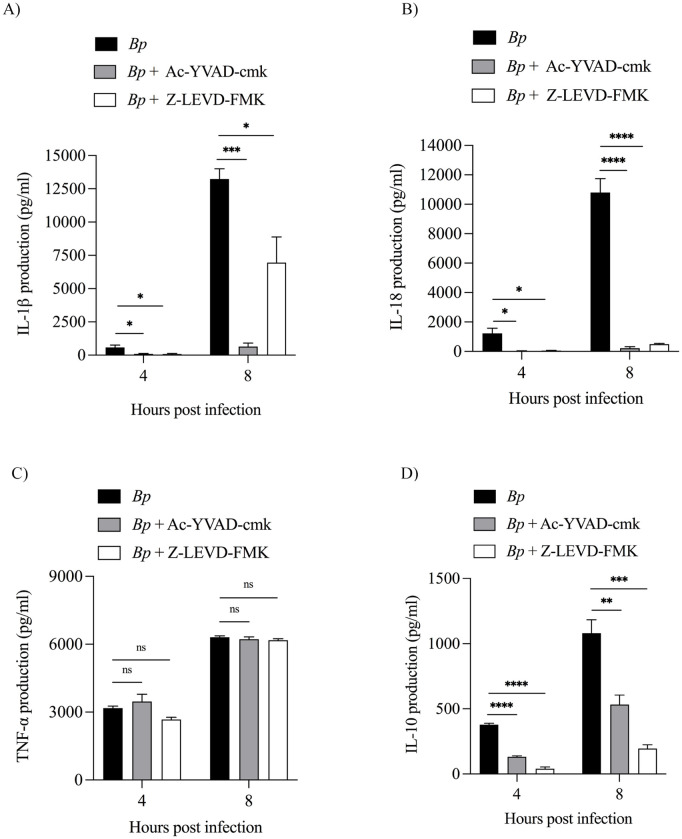
Attenuation of cytokine production by caspase inhibitors. (A-D) U937 macrophages were pretreated with 25 μM Ac-YVAD-cmk (caspase-1 inhibitor) for 30 min or 10 μM Z-LEVD-FMK (caspase-4 inhibitor) for 1 h before infection with *B. pseudomallei* at an MOI of 10. At the indicated time points, the level of cytokine secretion was determined by ELISA. Data are expressed as mean ± SEM from three independent experiments. One-way ANOVA followed by Tukey’s multiple comparison test was used to compare cytokine production. *, *P* < 0.05, **, *P* < 0.01, ***, *P* < 0.001, ****, *P* < 0.0001, ns = not significant.

We further demonstrated the effect of IL-1β and IL-18 production by using caspase-1 inhibitor (Ac-YVAC-cmk) and caspase-4 inhibitor (Z-LEVD-FMK). As shown in Fig 5A and 5B, both IL-1β and IL-18 levels were completely abolished at 4 and 8 hpi in Ac-YVAC-cmk-treated cells. In contrast, in the presence of Z-LEVD-FMK, both cytokine production was diminished at 8 hpi (Fig 5A and 5B). These results confirm the specific function of caspase inhibitors to dampen the release of inflammatory factors IL-1β, IL-18 and IL-10 but not TNF-α in *B. pseudomallei-*infected U937 macrophages (Fig 5C and 5D).

### ECDD-S16 does not significantly interfere with intracellular *B. pseudomallei* survival in U937 macrophages

**Fig 6:**
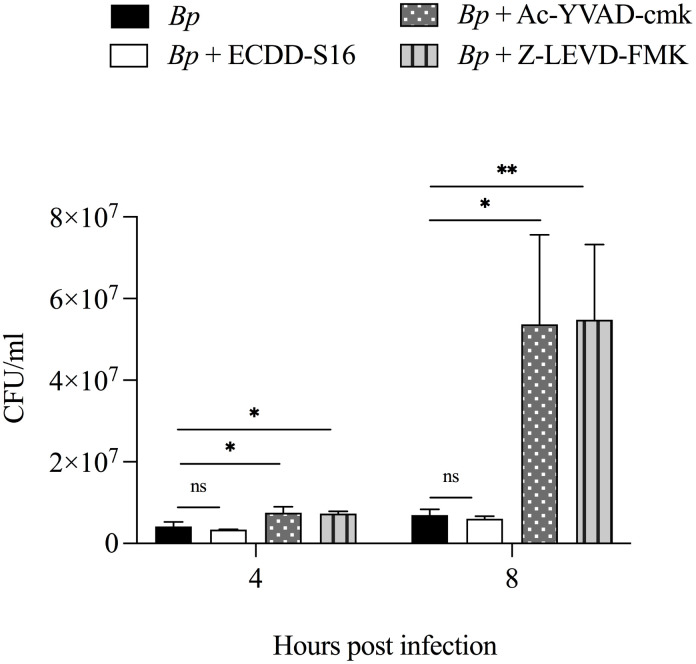
ECDD-S16 does not interfere with the intracellular survival of *B. pseudomallei* in U937 macrophages. U937 macrophages were infected with *B. pseudomallei* at an MOI of 10 in the presence of ECDD-S16, Ac-YVAD-cmk or Z-LEVD-FMK. At the indicated time points, the infected cells were lyzed and the number of intracellular bacteria was determined by plating for CFU. Data are mean ± SEM from three independent experiments. One-way ANOVA followed by Tukey’s multiple comparison test was used to compare CFU data. *, *P* < 0.05, **, *P* < 0.01, ns = not significant.

Numerous studies reported that inflammasome-mediated induction of pyroptosis could restrict growth and dissemination during *B. pseudomallei* infection [14,15]. Therefore, the pyroptosis reduction by ECDD-S16 may also interfere with intracellular replication of *B. pseudomallei.* To determine this, the intracellular replication of *B. pseudomallei* was examined in the presence or absence of ECDD-S16 treatment. As shown in Fig 6, compared to that in the absence of ECDD-S16, the number of intracellular *B. pseudomallei* survival in U937 macrophages was slightly decreased at 4 and 8 hpi. In contrast, the intracellular replication at 8 hpi was significantly increased in the presence of caspase-1 and caspase-4 inhibitors when compared to that seen in the untreated cells. The results suggested that ECDD-S16 could inhibit pyroptosis-induced inflammation without substantially interfering with intracellular *B. pseudomallei* survival in U937 macrophages.

### ECDD-S16 attenuates phagolysosome acidification

**Fig 7:**
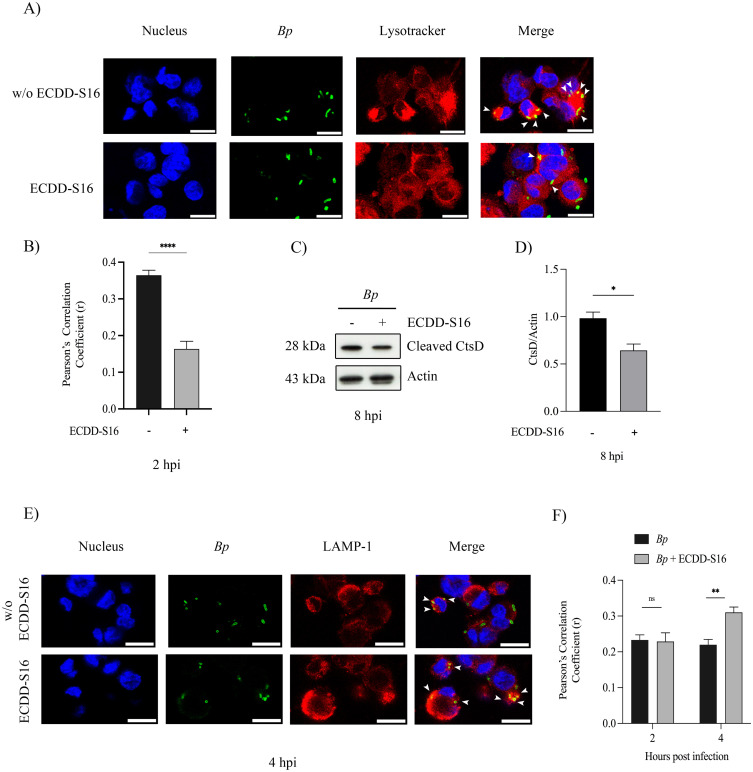
ECDD-S16 restricts *B. pseudomallei* by interfering with phagosome acidification. U937 macrophages were infected with *B. pseudomallei* at an MOI of 10 for 1 h before treatment with 1 μM ECDD-S16. (A) Representative image of confocal micrographs of cells infected with *B. pseudomallei* with or without ECDD-S16 treatment stained with anti-*B. pseudomallei* (green), Lysotracker (red) and Hoechst (blue). Scale bar is 20 μm. (B) Quantitative analysis of Lysotracker colocalization with *B. pseudomallei*. (C-D) One representative image of at least three independent experiments is shown for Western blotting (**C**) and quantitative analysis of CtsD/Actin by ImageJ (D). (E) Representative image of *B. pseudomallei-*infected control (untreated) and ECDD-S16-treated cells for 4 h and stained with anti-LAMP-1 antibody (red), anti-*B. pseudomallei* antibody (green) and Hoechst (blue). Scale bar is 20 μm. (F) Quantitative analysis of LAMP-1 association with *B. pseudomallei*. Data are mean ± SEM from three independent experiments. The Student’s t-test was used to compare. *, *P* < 0.05, **, *P* < 0.01, ****, *P* < 0.0001, ns = not significant.

It is well-documented that pyroptosis is involved in bacterial killing. The dampening of pyroptosis is usually due to increased intracellular bacteria [15]. Therefore, ECDD-S16 might suppress pyroptosis and have another role that can enhance macrophage killing. A previous study demonstrated that ECDD-S16 could interfere with vacuolar ATPase, decreasing phagolysosome acidification in RAW 264.7 cells activated with TLR ligands [13]. The acidification of phagosomes is essential for *B. pseudomallei* to escape into the cytosol, which leads to pathogenesis [16]. In this study, the colocalization between Lysotracker dye and *B. pseudomallei* was markedly decreased in ECDD-S16 treated cells when compared to that of the untreated control, suggesting that this compound prevented *B. pseudomallei*’s phagosome acidification (Fig 7A and 7B). Moreover, cathepsin D maturation is used as one of the markers to detect lysosome acidification [17]. Our results also demonstrated that ECDD-S16 significantly attenuated the amount of the cleaved form of cathepsin D, indicating that ECDD-S16 interfered with lysosome acidification in *B. pseudomallei*-infected U937 macrophages (Fig 7C and 7D). Moreover, in the presence of ECDD-S16, the colocalization of *B. pseudomallei* and LAMP-1 was significantly increased at 4 hpi, indirectly suggesting that the bacteria may be trapped within phagolysosome and cannot escape into the cytosol (Fig 7E and 7F). Therefore, ECDD-S16 can inhibit phagosome acidification, leading to the restriction of *B. pseudomallei* in LAMP-1- positive phagosomes.

## Discussion

Pyroptosis is well established as a pro-inflammatory form of programmed cell death that plays a pivotal role in host immune defense against invading pathogens [18]. This mechanism is triggered by activation of caspase-1 and caspase-4/5 (or caspase-11 in mice), leading to GSDMD cleavage, resulting in cell membrane rupture, release of inflammatory mediators, and cell death. This inflammatory cell death involves pro-inflammatory cytokine secretion and is essential in eliminating pathogens [18]. For example, GSDMD, a product of caspase-1/4/5 activation, can directly lyze extracellular bacteria such as *Escherichia coli* or *Staphylococcus aureus* by binding to cardiolipin (a lipid found in the bacterial cell membrane) and oligomerize to form pore on the bacterial surface [19–21]. Moreover, deficiency of GSDMD showed a significant increase in intracellular *Burkholderia* species growth compared to that of the control group [22]. Furthermore, mice deficient in caspase-1 were also more susceptible to infection with *B. pseudomallei* than the wild-type mice [15]. These results indicated that pyroptosis plays a critical protective role during bacterial infection.

Although induction of pyroptosis serves as a host defense to eliminate pathogens, excessive pro-inflammatory mediator production can lead to sepsis, which plays a significant cause in the mortality of melioidosis patients [15]. Therefore, preventing sepsis by suppressing pyroptosis without increasing bacterial replication may benefit the treatment of melioidosis, leading to an increase in lifespan. Previously, our group demonstrated that the decrease of LDH release and inflammatory caspase production (caspase-11) were observed in mouse macrophage cell line (RAW 264.7) that were pretreated with ECDD-S16 before activation with TLR ligands, suggesting the efficacy of this compound in suppression of pyroptosis [13]. In the present study, we further demonstrated the inhibitory effect of ECDD-S16 on pyroptosis in U937 macrophages infected with *B. pseudomallei*. This compound effectively attenuated pyroptosis activation by inhibiting caspase-1/4/5 and GSDMD activation in *B. pseudomallei-*infected U937 macrophages. Of note, the maturation of both IL-1β and IL-18, used as a marker for pyroptosis, was also inhibited in the presence of ECDD-S16. Similarly, blocking caspase activation using Ac-YVAC-cmk (caspase-1 inhibitor) and Z-LEVD-FMK (caspase-4 inhibitor) was found to reduce LDH release, implying that these inflammatory caspases participated in the induction of pyroptosis via the release of LDH. Decreased IL-1β and IL-18 production were also detected upon treatment of *B. pseudomallei*-infected cells with these caspase inhibitors, suggesting that activation of caspase-1/4 played a vital role in the regulation of pyroptosis-induced inflammation. These results are consistent with a previous report, which showed that Z-VAD-FMK (a caspase-1 inhibitor) could significantly decrease the secretion of IL-1β in patients with *S. aureus*-induced sepsis by inhibiting caspase activity [23]. Besides IL-1β and IL-18, both caspase inhibitors can also interfere with IL-10 production, consistent with previous reports in caspase-1/11-deficient mice infected with *Trypanosoma cruzi* [24]. In contrast, our study showed that ECDD-S16 could only suppress IL-1β and IL-18 but failed to inhibit IL-10 production. These results implied that ECDD-S16 may interfere with the cell signaling pathway in a different fashion compared to the caspase-1/4 activation in *B. pseudomallei*-infected U937 macrophages.

Generally, pyroptosis plays an important role in bacterial killing [15]. For example, deficiency of caspase-11 in mouse macrophages showed a higher number of intracellular *Legionella pneumophila* than that seen in the wild-type mice, suggesting the role of caspase-11 in inhibiting intracellular bacterial replication [25]. Additionally, caspase-1 and caspase-11 deficient mice are more susceptible to infection with *B. pseudomallei* or the closely related *B. thailandensis* [26]. Consistent with our findings in the presence of both caspase inhibitors, the attenuation of pyroptosis was observed, leading to increased *B. pseudomallei*’s intracellular survival in the human macrophage cell line. In contrast to both caspase inhibitors, ECDD-S16 reduced pyroptosis, but intracellular bacterial replication was not increased. Instead, the number of intracellular bacteria decreased slightly (not significantly) in *B. pseudomallei*-infected U937 macrophages. These results led us to believe that ECDD-S16 may have two roles in *B. pseudomallei*-infected U937 macrophages: one suppressed pyroptosis via caspase-1/4/5 and another reduced intracellular bacterial survival Furthermore, we investigated the effect of combining ECDD-S16 with antibiotics on *B. pseudomallei* survival. In vitro, ECDD-S16 alone did not significantly reduce bacterial viability compared to the untreated control. While the combination with ceftazidime significantly decreased bacterial counts. (S1 Fig). Similarly, the intracellular bacteria survival in *B. pseudomallei*-infected U937 cells was significantly suppressed in the ceftazidime treatment when compared to untreated cells (S2 Fig).

Previously, our group demonstrated that ECDD-S16 could inhibit pyroptosis by binding to the vacuolar (V-) ATPase, reducing phagosome acidification in RAW 264.7 cells activated by treatment of cells with TLR ligands [13]. Since phagosome acidification plays an essential role in bacterial virulence protein expression T6SS-1 and T3SS-3 [16]. These virulence factors facilitate *B. pseudomallei* to escape from the phagosome and thus can multiply inside the cytosol. Neutralize acidic organelles by chloroquine showed the suppression of intracellular growth of *B. thailandensis* and this bacterium was retained in the phagosome, indicating that evasion of this bacterium from the phagosome into the cytoplasm via bacterial virulence proteins requires acidic organelles [16]. In this study, we also demonstrated that ECDD-S16 could suppress phagosome acidification which might lead to the restriction of *B. pseudomallei* inside the phagosome and prevented phagosomal escape and thus intracellular multiplication inside the host cells. Therefore, using the combination of this compound together with antibiotics may be helpful for melioidosis treatment. However, this study has limitations due to its investigation in an *in vitro* model. It would be beneficial to explore the mechanisms of ECDD-S16 and its combinatorial effects with antibiotics for the treatment of melioidosis in an *in vivo* study, providing valuable insights into its potential therapeutic applications.

## Conclusion

Inflammation is one of the key host defenses against bacterial infection. Hence, giving anti-inflammatory drugs is not practical for treatment in patients with sepsis because suppression of inflammation would lead to an increase in the number of bacteria. However, in this study, we demonstrated the molecular mechanism of ECDD-S16 to reduce *B. pseudomallei*-induced pyroptosis without interfering with intracellular bacterial survival in U937 macrophages. Therefore, ECDD-S16 may be a candidate therapeutic compound for treating sepsis progression in acute melioidosis patients.

## Supporting information

S1 Fig Treatment of *B. pseudomallei* in vitro. Mid-log phase *B. pseudomallei* was treated with 10 mg/ml ceftazidime (CAZ) and 1 μM ECDD-S16 at different times. The number of viable bacteria was determined by OD650 measurement. Data are mean ± SEM from three independent experiments. One-way ANOVA followed by Tukey’s multiple comparison test was used to compare the OD650 measurement data. **, P < 0.01, ns = not significant.
(TIF)

S2 Fig ECDD-S16 does not synergize with ceftazidime to inhibit the intracellular survival of *B. pseudomallei* in U937 macrophages. U937 macrophages were infected with *B. pseudomallei* at an MOI of 10 for 1 hour before adding 1 μM ECDD-S16 and 10 mg/ml ceftazidime (CAZ). At 8 hours post-infection, the infected cells were lyzed and the number of intracellular bacteria was determined by plating for CFU. Data are mean ± SEM from three independent experiments. One-way ANOVA followed by Tukey’s multiple comparison test was used to compare CFU data. ***, P < 0.001, ns = not significant.
(TIF)

S1 Data The numerical data used in all figures are included in S1 Data.xlsx.
(XLSX)
