# Supplementary figures and images for: ECDD-S16, a synthetic derivative of cleistanthin A, suppresses pyroptosis in Burkholderia pseudomallei-infected U937 macrophages
Source: PLoS One. 2025 Jul 8;20(7):e0327457. doi: 10.1371/journal.pone.0327457 (PMC12237264; doi:10.1371/journal.pone.0327457)

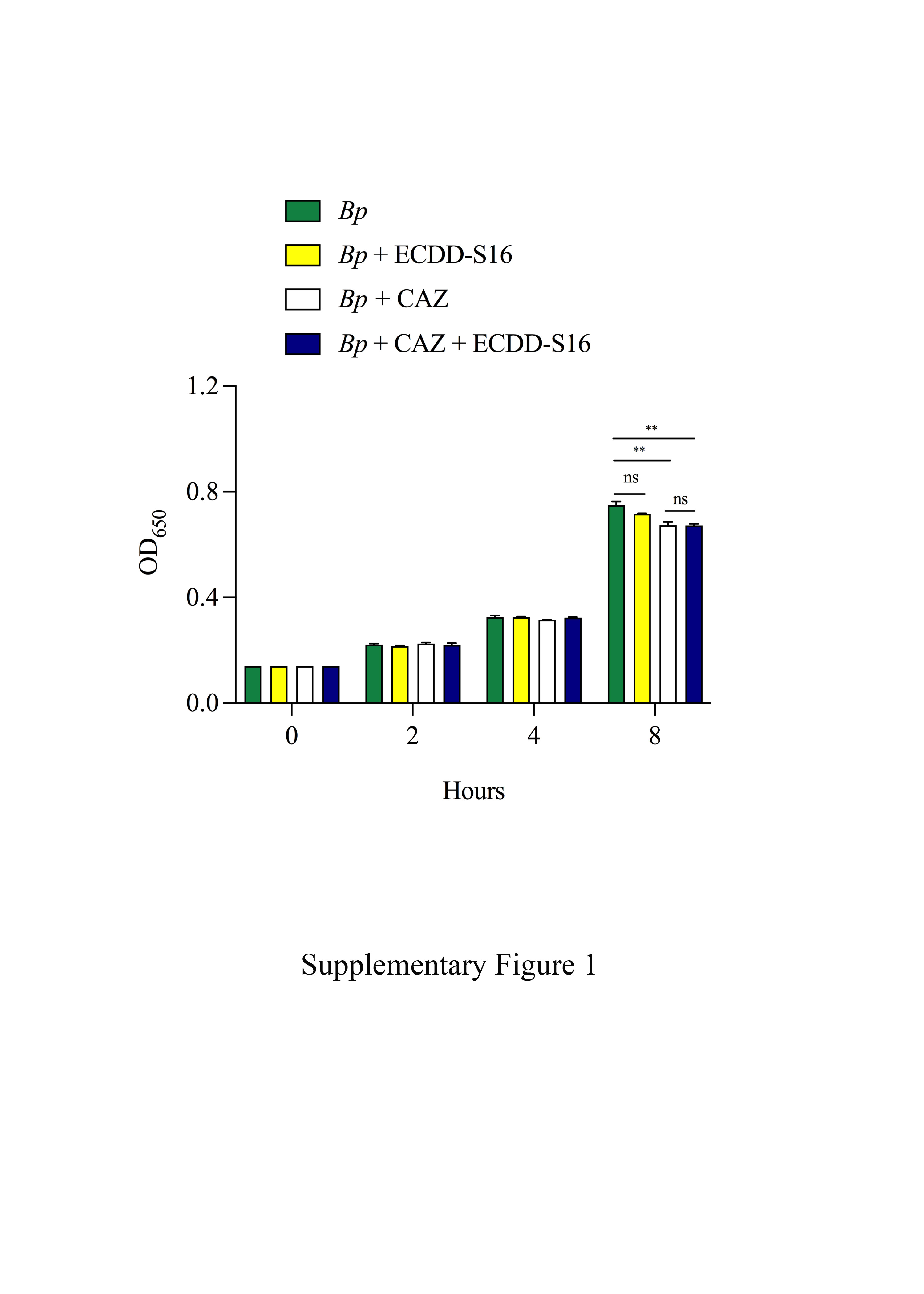

Supplement: S1 Fig — Mid-log phase B. pseudomallei was treated with 10 mg/ml ceftazidime (CAZ) and 1 μM ECDD-S16 at different times. The number of viable bacteria was determined by OD650 measurement. Data are mean ± SEM from three independent experiments. One-way ANOVA followed by Tukey’s multiple comparison test was used to compare the OD650 measurement data. **, P < 0.01, ns = not significant. (TIF) [file pone.0327457.s001.tif]

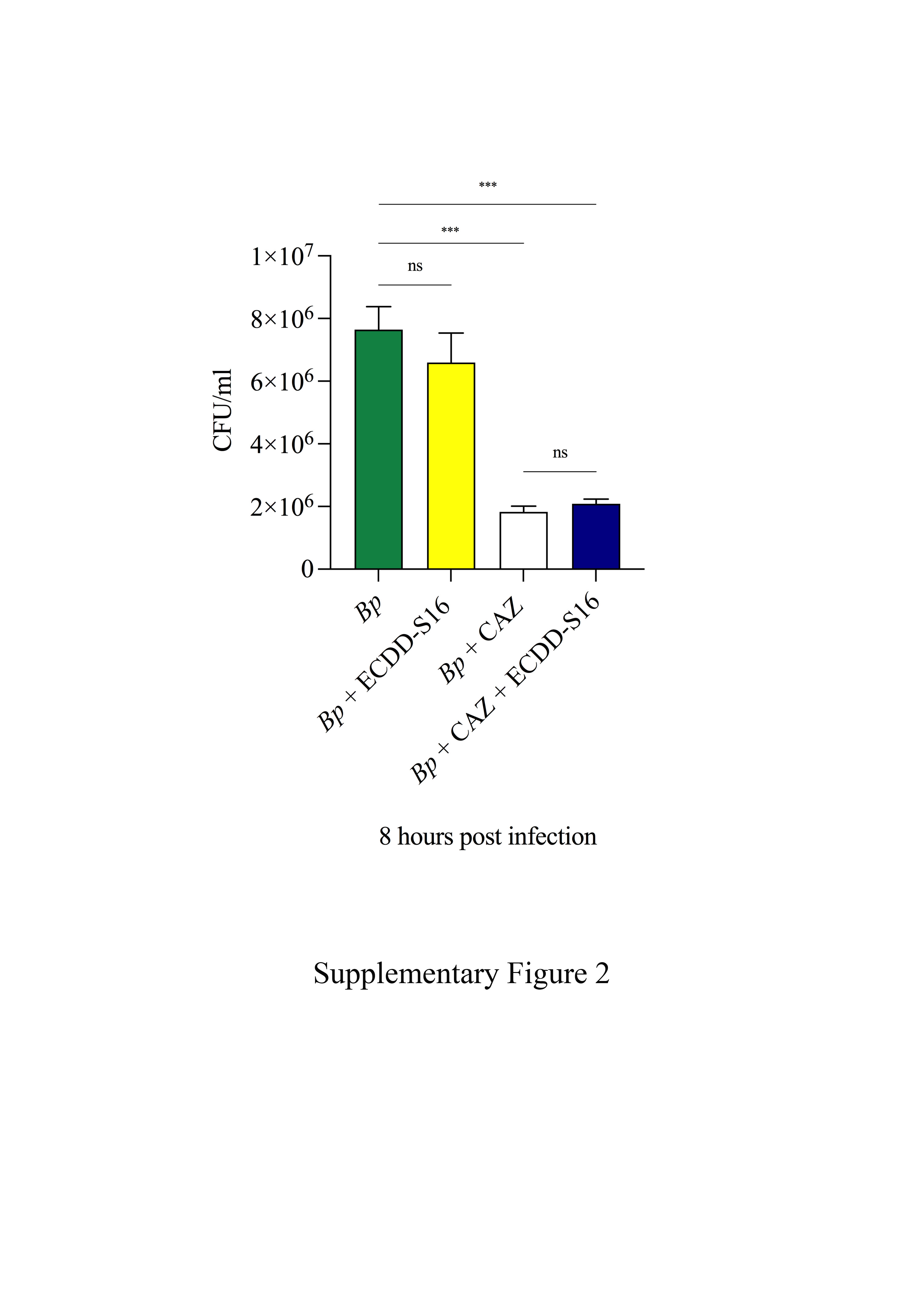

Supplement: S2 Fig — U937 macrophages were infected with B. pseudomallei at an MOI of 10 for 1 hour before adding 1 μM ECDD-S16 and 10 mg/ml ceftazidime (CAZ). At 8 hours post-infection, the infected cells were lyzed and the number of intracellular bacteria was determined by plating for CFU. Data are mean ± SEM from three independent experiments. One-way ANOVA followed by Tukey’s multiple comparison test was used to compare CFU data. ***, P < 0.001, ns = not significant. (TIF) [file pone.0327457.s002.tif]
